# Fibrinogen and Prothrombin Complex Concentrate: The Importance of the Temporal Sequence—A Post-Hoc Analysis of Two Randomized Controlled Trials

**DOI:** 10.3390/jcm13237137

**Published:** 2024-11-25

**Authors:** Marco Ranucci, Tommaso Aloisio, Umberto Di Dedda, Martina Anguissola, Alessandro Barbaria, Ekaterina Baryshnikova

**Affiliations:** Department of Cardiothoracic and Vascular Anesthesia and Intensive Care, Istituto di Ricovero e Cura a Carattere Scientifico, Policlinico San Donato, San Donato Milanese, 20097 Milan, Italy; tommaso.aloisio@grupposandonato.it (T.A.); umberto.didedda@grupposandonato.it (U.D.D.); martina.anguissola@grupposandonato.it (M.A.); alessandro.barbaria@grupposandonato.it (A.B.); ekaterina.baryshnikova@grupposandonato.it (E.B.)

**Keywords:** prothrombin complex concentrate, fibrinogen, cardiac surgery, postoperative bleeding, viscoelastic tests, thromboelastometry

## Abstract

**Background/Objectives**: A low level of soluble coagulation factors after cardiac surgery may cause excessive bleeding and trigger clinical correction using prothrombin complex concentrate (PCC). According to the current guidelines, the trigger values for PCC administration are not defined. In the published algorithms, when driven by ROTEM^®^, the triggers vary from 80 s to >100 s of coagulation time (CT) during an EXTEM test. Two randomized controlled trials on fibrinogen (FC) supplementation after cardiac surgery previously pointed out that the patients receiving FC supplementation had a significant decrease in their EXTEM CT. This study investigates the hypothesis that after increasing the availability of a substrate (fibrinogen), thrombin generation induces fibrin network formation faster, and that, before considering PCC administration, the normalization of fibrinogen levels should be sought. **Methods**: A retrospective study based on a post-hoc analysis of the data collected in two previous RCTs involving 85 patients, all of whom received FC supplementation. **Results**: The results of this post-hoc analysis demonstrate that there is a significant negative association between FIBTEM maximum clot firmness (MCF) and the EXTEM CTs before and after FC supplementation; FC supplementation decreases the EXTEM CTs both in patients with a low FIBTEM MCF and a normal FIBTEM MCF. After FC supplementation, 45 (53%) of the patients had an EXTEM CT of >80 s, 22 (26%) had an EXTEM CT of >90 s, and 8 (9%) had an EXTEM CT of >100 s. **Conclusions:** Our study confirms and quantifies the effects of reducing EXTEM CTs through FC supplementation. A stepwise strategy of factors correction with FC supplementation should be used before considering PCC administration as it might reduce the need for PCC.

## 1. Introduction

Postoperative bleeding after cardiac surgery with cardiopulmonary bypass (CPB) is still a common clinical problem. The causes of coagulopathy include (a) residual heparin, (b) thrombocytopenia and/or platelet dysfunction, (c) hypofibrinogenemia, (d) low levels of soluble coagulation factors, and (e) hyperfibrinolysis [1,2,3,4,5,6,7]. To tackle the diagnosis of and therapy for coagulopathic bleeding, the existing guidelines [8] provide a class I recommendation for the use of specific bleeding treatment algorithms. Fibrinogen concentrate (FC), and prothrombin complex concentrate (PCC) are possible therapeutic options to correct coagulopathic bleeding, and both FC and PCC are suggested with a recommendation class IIa. However, a specific trigger value for FC supplementation is indicated (<1.5 g/L) whereas for PCC the only trigger is “coagulation factors deficiency”, without precise values.

Many bleeding management algorithms have been published in the setting of postoperative bleeding after cardiac surgery, including the possible point-of-care (POC)-based trigger values for PCC administration. These trigger values range from a coagulation time (CT) during the ROTEM^®^ EXTEM (Tem Innovations GmbH, Munich, Germany) test (EXTEM CT) of >80 s [9,10,11] to a CT time of >90 s [12,13] or up to a time of >100 s [14,15,16,17]; this last trigger value is suggested by a document from the Society of Cardiovascular Anesthesiologists [17].

During our randomized controlled trial (RCT) for FC supplementation [9], we noticed and reported that patients receiving FC supplementation, besides an obvious increase in FIBTEM maximum clot firmness (MCF), had a significant decrease in their EXTEM CTs. This raises the question of whether increasing the concentration of a substrate (fibrinogen) enables thrombin generaion to form the fibrin network at a higher speed. Therefore, before considering PCC administration, the normalization of fibrinogen values is recommended.

The present study investigates the hypothesis that increasing clot firmness through FC supplementation is associated with a concomitant shortening of the reaction time (CT). We investigated this hypothesis through a post-hoc analysis of two previous RCTs [9,18] performed by our group and based on FC supplementation. The potential clinical relevance of this hypothesis is based on the evidence that different clinical conditions are burdened by acquired hypofibrinogenemia and bleeding. Among these clinical conditions, and apart from cardiac surgery, are the post-partum hemorrhage [19], liver surgery [20], trauma [20], and others. In general, acquired hypofibrinogenemia may be due to a reduced synthesis or an increased consumption of clotting factors; as such, liver dysfunction and extreme coagulation activation are the main causes of this condition.

## 2. Materials and Methods

### 2.1. Study Design

A post-hoc analysis of the data collected in two previous RCTs.

### 2.2. Patient Population

For the purposes of the present study, we retrieved the rough data of the ZEro Plasma Trial (ZEPLAST, 2015) [9] and of the Effectiveness of DIfferent fibrinogen PreparatiOns in REstoring clot firmness (EDIPO RE, 2024) [18] trial. Both the studies were RCTs approved by the Ethics Committee of ASL Melegnano (EudraCT code 2011-0046-33) and San Raffaele Hospital (approval number 129/INT/2022). The EDIPO RE included only the patients (N = 40) with low (<10 mm) FIBTEM MCF values after CPB, comparing a dose of about 30 mg/kg of FC from two different companies, in terms of the FIBTEM MCF increase. Within this protocol, an EXTEM test performed at the same time as a ROTEM test was included; therefore, the EXTEM CT values were available pre- and post-FC supplementation, and all the patients from the EDIPO RE trial were included in the present study. The ZEPLAST compared the clinical outcomes of 120 patients who were randomized to receive FC supplementation or a placebo, regardless of the FIBTEM MCF values. In this study, large doses of FC were used in order to reach the goal of a FIBTEM MCF of 22 mm. Forty-five patients in the FC arm had EXTEM data available before and after FC supplementation, and were included in the present study. Therefore, the total patient population is represented by 85 patients, all receiving FC supplementation, with ROTEM data available before and after the supplementation. In both studies, ROTEM Delta was used.

### 2.3. Data Collection

The following data were retrieved: the weight of the patient; FC dose (mg and mg/kg); and EXTEM CT and FIBTEM MCF before and after FC supplementation.

### 2.4. Statistics

The differences pre- and post-FIBTEM MCF and CT were analyzed with a paired Student’s *t* test.

The relationship between the FIBTEM MCF and EXTEM CT was investigated separately before and after FC supplementation, using linear regression analyses, producing a linear equation with 95% confidence intervals.

Linear and polynomial regression analyses were applied to investigate the relationship between a FIBTEM MCF increase and an EXTEM CT decrease, producing, for the best-fit equation, 95% confidence intervals. Finally, the effects of FC supplementation were expressed in terms of a binary classification of the patients according to the commonly used cut-values of EXTEM CTs (80, 90, and 100 s), in order to establish the potential need for PCC administration before and after FC supplementation.

All the statistical analyses were performed with computerized packages (SPSS 20.0, IBM, Chicago, IL, USA; and GraphPad, GraphPad Software 10.0.2, Boston, MA, USA). A *p* value of <0.05 was considered significant for all the statistical tests.

## 3. Results

The general characteristics of the patient population are presented in Table 1. Overall, 85 patients are included (40 patients from the EDIPO RE trial, and 45 from the treatment arm of the ZEPLAST) with ROTEM data before and after FC supplementation.

**Figure 1:**
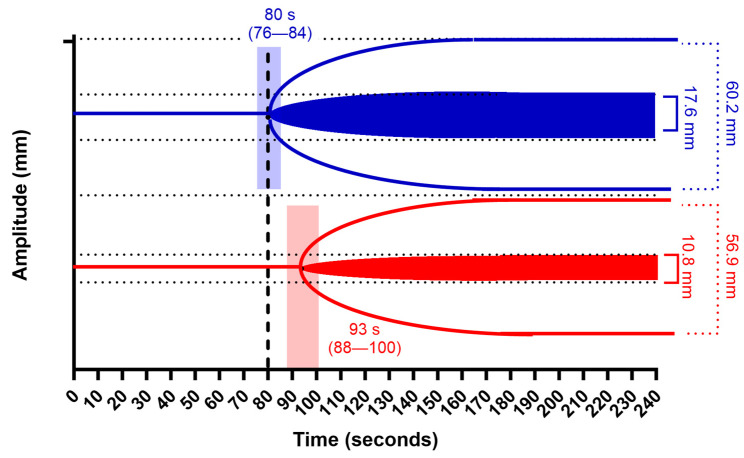
Graphical representation of ROTEM (EXTEM + FIBTEM) changes before (red) and after (blue) fibrinogen concentrate supplementation. Data are the mean, with a 95% confidence interval (transparent rectangles) for EXTEM clotting time (CT). Dashed line is the upper limit of the EXTEM CT normal range.

The patients received different FC doses according to the different protocols of the two RCTs. In the EDIPO RE trial, the dose was set to 30 mg/kg, with an approximation value of 2 or 3 g, in the patients with a post-protamine FIBTEM MCF of <10 mm. Conversely, the patients in the ZEPLAST received a dose targeted to reach a FIBTEM MCF of 22 mm. Overall, the dose varied from 1 g to 11 g (11 to 92 mg/kg). The mean increase of FIBTEM MCF was 6.9 mm (*p* = 0.001) with a correspondent increase of EXTEM MCF of 3.3 mm (*p* = 0.001). The EXTEM CT had a concomitant mean decrease of 13.1 s (*p* = 0.001). A graphical representation of the changes in the ROTEM tests (EXTEM and FIBTEM) before and after FC supplementation and based on the mean values with a 95% confidence interval is presented in Figure 1.

**Figure 2:**
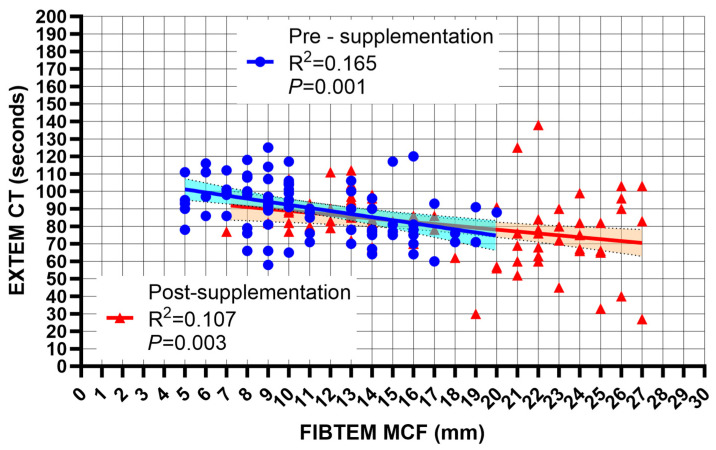
Linear regression analysis of EXTEM CT (clotting time) as a function of FIBTEM MCF (maximum clot firmness) before and after fibrinogen supplementation. CT: clotting time; MCF: maximum clot firmness.

**Figure 3:**
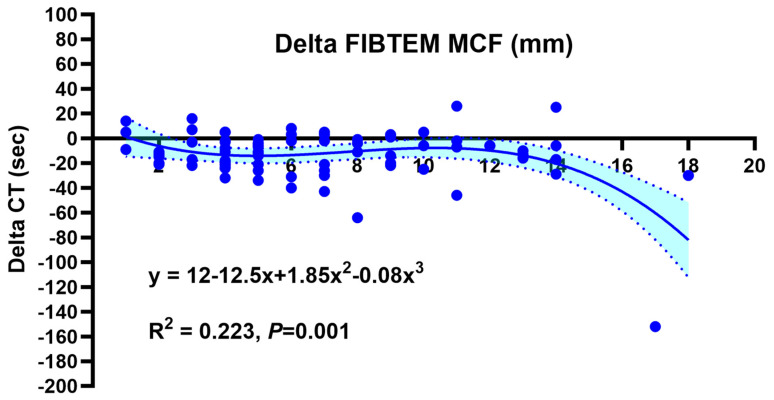
Cubic regression analysis of EXTEM CT (clotting time) decreases as a function of FIBTEM MCF (maximum clot firmness) increases after fibrinogen supplementation. CT: clotting time; MCF: maximum clot firmness.

There was a significant negative correlation between the FIBTEM MCF values and EXTEM CT values both before (*p* = 0.001) and after (*p* = 0.003) FC supplementation (Figure 2), with a similar slope of linear regression (with an EXTEM CT decrease of 1.4 s per unit increase [mm] of the FIBTEM MCF). The effects of FC supplementation on EXTEM CTs are shown in Figure 3. Seventy (82%) patients showed a post-supplementation EXTEM CT decrease, and the extent of a FIBTEM MCF increase was significantly (*p* = 0.001) associated with an EXTEM CT decrease according to a cubic regression analysis.

Given the different protocols for FC supplementation in the two RCTs, we performed a sub-analysis comparing the ROTEM tests in the two studies. Before FC supplementation, the CT at EXTEM was significantly (*p* = 0.016) shorter in the ZEPLAST than in the EDIPO RE (86 ± 35 s vs. 100 ± 12 s) and the FIBTEM MCF was significantly (*p* = 0.001) higher (13 ± 4 mm vs. 8 ± 1.7 mm). However, the decrease in EXTEM CT after FC supplementation did not differ between the ZEPLAST (−13 ± 27 s) and the EDIPO RE trial (−13 ± 12 s).

**Figure 4:**
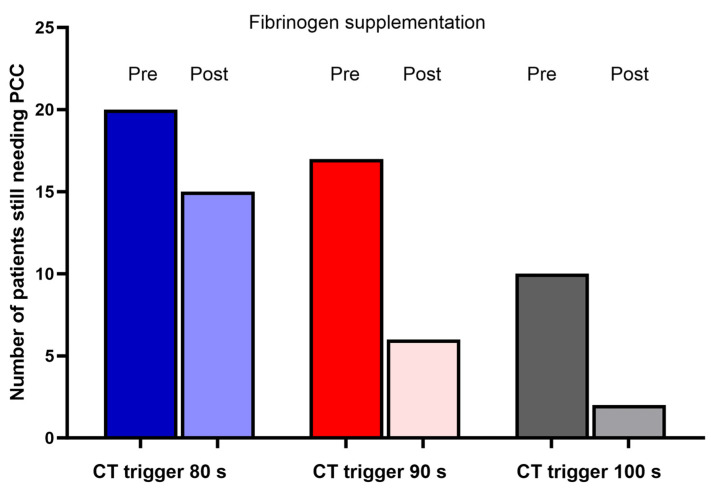
Number of patients with clotting times (CTs) longer than the trigger value for prothrombin complex concentrate administration at 3 different trigger values. Analysis restricted to patients with low fibrinogen levels, a FIBTEM MCF (maximum clot firmness) of <10 mm.

The practical effects of these analyses, and namely of the EXTEM CT decrease subsequent to FC supplementation, have been investigated based on the three different EXTEM CT values that are considered possible triggers for PCC administration by the existing guidelines/algorithms: 80 s, 90 s, and 100 s (Figure 4). For each cut-off value, the number of patients who would have received PCC was calculated and compared with the number who would not receive PCC after FC supplementation, within the patients with a low (<10 mm) FIBTEM CT after protamine administration (N = 40). For a trigger value of >80 s, 20 patients (50%) would have received PCC before FC supplementation, and 15 (38.4%) would be still in need of PCC after FC supplementation (a relative decrease of 25%); for a trigger value of >90 s, 17 patients (43.6%) would have received PCC before FC supplementation, and 6 (15.4%) would be still in need of PCC after FC supplementation (a relative decrease of 65%). Finally, for a trigger value of >100 s, 10 patients (25.6%) would have received PCC before FC supplementation, and only 2 (5.1%) would be still in need of PCC after FC supplementation (a relative decrease of 80%). Overall, in the whole patient population, using a trigger value > 100 s and after FC supplementation, only 2.3% of the patient population would still require PCC.

Forty-five patients received FC supplementation even if the FIBTEM MCF was ≥10 mm. In this patient population, nine (20%) subjects had an EXTEM CT of >100 s before FC supplementation, and only four (8.8%) had this after FC supplementation, with a relative decrease of 56%.

Overall, in the total patient population, only eight (9%) patients maintained an EXTEM CT of >100 s after FC supplementation.

## 4. Discussion

The results of this post-hoc analysis of two RCTs clearly demonstrate that (i) there is a significant negative association between FIBTEM MCF and EXTEM CT, that is almost identical before and after FC supplementation; and (ii) FC supplementation decreases the EXTEM CT, both in patients with a low FIBTEM MCF and, even if to a lesser degree, in those with a normal FIBTEM MCF. After FC supplementation, 45 (53%) of the patients had an EXTEM CT of >80 s, 22 (26%) had an EXTEM CT of >90 s, and 8 (9%) had an EXTEM CT of >100 s.

There are methodological differences between the two studies analyzed in this manuscript. The first is that in the ZEPLAST the ROTEM sample was drawn at the end of a CPB performed under full heparinization, whereas in the EDIPO RE trial this was performed after a heparin reversal with protamine. The second is that, in the ZEPLAST, neither microvascular bleeding nor low fibrinogen levels were needed to receive FC. The third is that, in the ZEPLAST, large doses of FC aimed at reaching a FIBTEM MCF of 22 mm were used, while in the EDIPO RE trial the dose was 30 mg/kg for all the patients. Not surprisingly the ROTEM values before FC supplementation were less altered in the ZEPLAST; however, it is worthwhile to notice that, regardless of this difference and the different FC doses, in both the ZEPLAST and the EDIPO RE trial the shortening of an EXTEM CT after FC supplementation was the same. This actually seems to demonstrate that regardless of the FC dose and the target FIBTEM MCF, fibrinogen normalization causes a similar shortening of an EXTEM CT.

Performing the ROTEM test under full heparinization (ZEPLAST) does not alter the FIBTEM MCF [21], but conflicting evidence exists for the EXTEM CT, with authors claiming a prolongation of the CT occurs with very high concentrations of heparin [22] or immediately after heparinization [23]. In our study, the EXTEM CT at the end of a CPB was shorter than after protamine administration, but this depends on the different methods for FC supplementation in the two RCTs.

PCC administration is suggested by the existing guidelines and algorithms in the cases of coagulopathic bleeding and prolonged reaction times during viscoelastic tests. However, there is no consensus about the CT trigger value that prompts PCC administration in a bleeding patient. The European guidelines [8] do not specify any specific value, and do not consider the sequence of interventions to apply in the presence of bleeding with both a fibrinogen deficiency and soluble coagulation factors deficiency (namely, the simultaneous presence of a low FIBTEM MCF and a prolonged EXTEM CT at time of the ROTEM test). This, actually, configures a sort of “horizontal” bleeding treatment algorithm. Conversely, other guidelines [17] suggest a “vertical” approach, with FC (or cryoprecipitate) supplementation employed before considering PCC administration. In an RCT [10], our group demonstrated that supplementation with a high-dose of FC resulted in a lower rate of allogeneic blood products transfusions and eliminated the need for fresh frozen plasma (FFP) and PCC administration. Similar results were obtained in a study [15] where a vertical algorithm, with FC supplementation before considering the EXTEM CT, resulted in 20% of patients receiving FC supplementation, but none received FFP or PCC. A large before-and-after study (including 1900 patients), using a bleeding treatment algorithm and considering FC supplementation before addressing reaction times, reduced the use of FFP from 12.8% to 5.4%, and the use of PCC from 13% to 9.7% without any increases in FC use [14]. However, in general, prioritizing FC supplementation with respect to other measures generally increases FC use: Kuiper and associates [13] demonstrated that the application of a vertical algorithm resulted in increased FC supplementation (16.8% vs. 8.9%) but a dramatic decrease in FFP use (from 18% to 6.9%) with no use of PCC occurring. The same result was obtained by Monaco and coworkers [11] in a series of patients undergoing aortic arch surgery: the use of a vertical algorithm with FC supplementation before FFP or PCC resulted in a higher rate of FC administration, with a concomitant decrease of FFP use (from 68% to 14%) but a higher use of PCC (from 5.3% to 29%). Finally, the seminal study of Weber and associates [10] applied a vertical algorithm based on FC supplementation before FFP/PCC administration. This resulted in a decrease in FFP use from 80% to 40%, and of PCC from 52% to 44%.

The horizontal algorithms produced different effects. A horizontal algorithm by Karkouti and associates [16] in a large (1170) patient population ended up with a combined administration of FFP, PCC, and rFVIIa of 25%. Of notice, only 5% of the patient population received fibrinogen supplementation (either cryoprecipitate or FC). The same group addressed a patient population of 7402 patients receiving cardiac operations in 12 Canadian hospitals [12]. The application of the same horizontal algorithm produced a reduction in red blood cell transfusion, but the effects on FC/cryoprecipitate supplementation and the use of FFP remained around 20% (no information on PCC).

Overall, the studies where FC supplementation is considered before FFP/PCC administration show a significant decrease in the use of pro-coagulants aimed at increasing thrombin generation and correcting soluble coagulation factors deficiency. Our study provides a justification for this finding, showing that actually prolonged CTs are very often just an epiphenomenon of a substrate (fibrinogen) deficiency, and that correcting substrate levels restores CTs to values that do not suggest the use of other pro-coagulants.

Of course, the extent of the potential limitation in the use of FFP and especially PCC depends on the trigger value that is applied. It is not the purpose of the present study to suggest which is the most appropriate EXTEM CT trigger value for PCC administration; it is, however, clear that the more restrictive (higher) it is, the more advantage there is for fibrinogen correction in terms of a reduction in PCC use.

Reducing PCC administration through a restrictive policy and, when needed, with a preliminary correction of low fibrinogen levels is a reasonable target, both from an economical perspective and especially in terms of safety features. PCC administration may result in an increased risk of thromboembolic events [24], and it should be used in small doses and within a strict algorithm [25,26]. Conversely, there are no studies or reports of increased thrombogenicity with FC supplementation.

In general, the concept of sequential interventions in a bleeding patient is the basis of vertical algorithms; its rationale is practical, clinical, and financial. In daily practice, residual heparin is a relatively common and easy-to-manage bleeding condition and is usually considered as the first option to investigate. Subsequently, platelets and fibrinogen should be considered. These are the real components of a stable clot (with the contribution of FXIII) and the targets of thrombin generation. Therefore, eliciting thrombin generation with PCC or rFVIIa is ineffective (and expensive) if the substrate is not adequate. Additionally, increasing thrombin generation may be responsible for thrombotic complications.

There are limitations in this study. It is a post-hoc analysis of two RCTs that were not designed to address the effect of fibrinogen supplementation on coagulation reaction times. This results in a lacking power analysis, and therefore, the sample size could be inadequate to address the experimental hypothesis. A possible selection bias could be that in the ZEPLAST the patients received FC regardless of their FIBTEM values, whereas in the EDIPO RE trial the use of FC supplementation was triggered by low levels of fibrinogen. Different doses of FC were used in the two studies, and different timing for the ROTEM analyses was applied. Finally, the level of association between a FIBTEM increase and a CT decrease is sub-optimal (R^2^ 0.165–0.223).

The results of our studies need to be replicated in a larger series, and possibly in other scenarios (trauma, post-partum hemorrhage, etc.). If confirmed, the use of PCC will probably decrease, providing advantages in terms of pharmaco-economics and safety.

## 5. Conclusions

In conclusion, our study confirms and quantifies the effects of reducing the EXTEM CT through FC supplementation. The practical consequence of a stepwise strategy of factors correction with FC supplementation before considering PCC administration is that it can significantly reduce the need for PCC, ultimately enhancing patient safety and optimizing resource utilization.

## Figures and Tables

**Table 1:** Fibrinogen supplementation and viscoelastic parameters pre- and post-supplementation (N = 85).

		Pre-Supplementation	Post-Supplementation	Mean Difference	*p*
Weight (kg)	74.9 (15.1)				
Fibrinogen dose (mg)	3541 (1893)				
Fibrinogen dose/kg (mg)	47.4 (23.0)				
FIBTEM MCF (mm)		10.8 (3.8)	17.6 (5.8)	6.9 (6.0 to 7.8)	0.001
EXTEM MCF (mm)		56.9 (9.9)	60.2 (5.7)	3.3 (1.6 to 4.8)	0.001
CT EXTEM (s)		93.1 (27.2)	80.1 (18.9)	−13.1 (−17.8 to −8.4)	0.001

Data in brackets are the standard deviation or 95% confidence interval.

## Data Availability

The original dataset supporting the findings of this study will be deposited in the public repository Zenodo after the publication of this paper and will be accessible upon a reasonable request to the corresponding author.
